# Evaluation of an Artificial Intelligence and Machine Learning Algorithm to Enhance Angiographic Coronary Visibility in Morbid Obesity: A Retrospective Observational Study

**DOI:** 10.1016/j.jscai.2026.105627

**Published:** 2026-08-31

**Authors:** Samia Mazumder, Tim Joseph, Devraj Sukul, Araya Negash, Justin Armstrong, Lakshmi Rao, Najeebullah Bangash, Ryan D. Madder

**Affiliations:** a Center for Innovation & Research in Cardiovascular Diseases; Department of Cardiovascular Medicine, William Beaumont University Hospital, Corewell Health East, Royal Oak, Michigan; b Frederik Meijer Heart & Vascular Institute, Corewell Health West, Grand Rapids, Michigan; c Michigan State University College of Human Medicine, Grand Rapids, Michigan

## Introduction

Invasive coronary angiography is an essential diagnostic tool to assess coronary artery disease but is often limited by suboptimal image quality among obese patients. A novel artificial intelligence (AI) and machine learning algorithm using spatiotemporal enhancement processing (STEP) has been developed to enhance angiographic images and improve coronary visualization,^1^^,^^2^ but whether STEP improves suboptimal image quality in morbidly obese patients has not been studied. This study was performed to evaluate whether STEP can enhance coronary visualization among cineangiograms acquired in morbidly obese patients.

## Materials and methods

In this single-center retrospective observational study, consecutive patients with body mass index ≥40 kg/m^2^ undergoing invasive coronary angiography were identified. Only angiograms performed using the maximum tube voltage (125 kV) were selected for analysis, since this indicates the fluoroscopic system’s automatic exposure control mechanisms were maximized in an attempt to maintain image quality. The local institutional review board approved the protocol and provided a waiver of consent.

Included cineangiograms were processed using STEP (AngioWaveNet, AngioWave Imaging), an advanced AI and machine learning algorithm employing a neural network architecture using spatial and temporal information from blocks of 5 contiguous frames to enhance vessels in the central frame. Standard and STEP cineangiograms were independently evaluated side-by-side by 4 cardiologists to grade respective image quality on a 5-point Likert scale (1 = nondiagnostic; 2 = poor; 3 = acceptable; 4 = good; 5 = excellent). Quantitative analyses of standard and STEP cineangiograms were performed by selecting 4 frames at random from each cineangiogram and computing contrast (quantifies relative differences in signal intensity), signal-to-noise ratio (SNR) (characterizes strength of signal relative to noise), and signal detectability (D′) (integrates signal and noise through statistical decision theory to predict how well an ideal observer can detect signal).^3^

The primary hypothesis was that STEP would improve vessel visibility. This was tested using a one-sided Wilcoxon signed-rank test to compare Likert scores between standard and STEP cineangiograms. Inter-reader agreement was assessed using Fleiss’ kappa. Contrast, SNR, and D′ were similarly compared between standard and STEP cineangiograms. All statistical analyses were performed using custom Python code (Python 3.13.3, NumPy 2.2.5, SciPy 1.15.2, Pandas 2.2.3).

## Results

Among 609 cineangiograms obtained in 50 consecutive patients with body mass index ≥40 kg/m^2^, 34 (5.6%) cineangiograms acquired at maximum tube voltage were identified in 23 patients and were included in the analysis. Improved coronary visualization, as indicated by a higher average Likert score of image quality, was observed with STEP in 23 of 34 cineangiograms (67.6 %). The mean and median per-cineangiogram deltas were +1.03 and +1.00, respectively. The overall Likert score increased from 2.90 ± 0.46 among standard cineangiograms to 3.22 ± 0.59 using STEP (*P* = .0005; Figure 1). To address within-patient correlation among cineangiograms, a patient-level cluster bootstrap (*B* = 20,000) was performed and yielded a one-sided *P* value of .007 (95% CI for the mean paired difference: +0.18 to +0.46 Likert points), confirming that the improvement with STEP remained statistically significant after accounting for the nonindependence of cineangiograms within patients. The number of individual reader ratings of “good” or “excellent” increased from 15.4% of standard cineangiograms to 37.5% among STEP cineangiograms (*P* < .001). Inter-reader agreement was moderate for standard cineangiograms (*κ* = 0.441) and fair for STEP cineangiograms (*κ* = 0.292). Among quantitative imaging metrics, contrast increased by a mean of 10.7% ± 4.3% (95% CI, 8.5%-12.5%, *P* < .001), mean D′ improved from 0.46 ± 0.33 to 0.91 ± 0.32 (D′ increase +0.45, 95% CI, 0.33-0.55, *P* < .001), and SNR improved from 0.42 ± 0.24 to 0.82 ± 0.29 (SNR increase +0.40, 95% CI, 0.31-0.48, *P* < .001).

**Figure 1:**
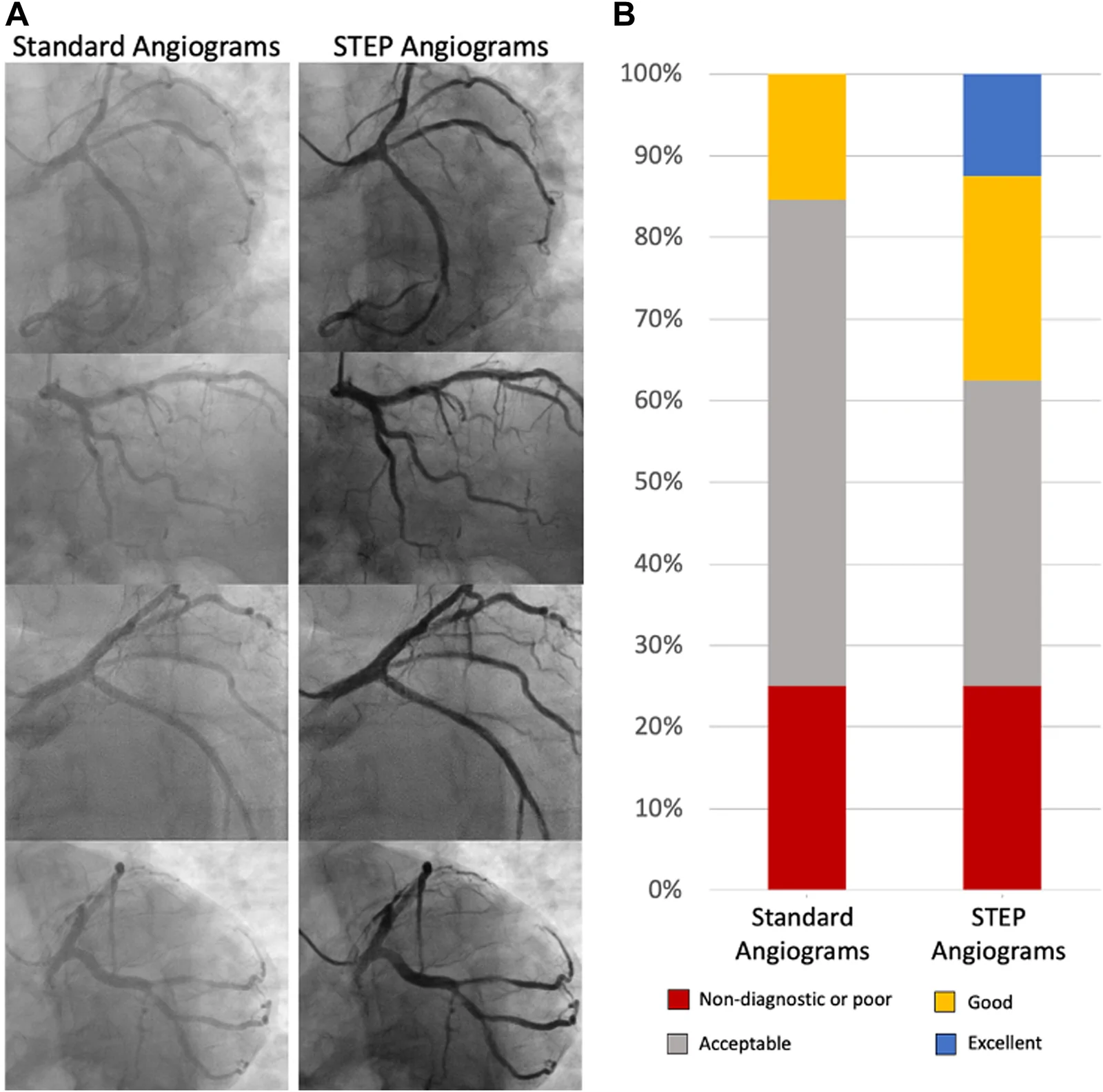
**Comparison of standard and STEP cineangiograms in morbidly obese patients.** Paired frames from standard and STEP angiograms (A) and expert reader image grading results (B) are shown. STEP, spatiotemporal enhancement processing.

## Discussion

Among cineangiograms acquired in morbidly obese patients, application of STEP was observed to significantly improve coronary visualization. STEP, which utilizes temporal information to distinguish pixels likely to be vessels from the rest of the image, was recently approved by the FDA with a label claim to enhance the visibility of coronary angiograms but had not been previously tested under the challenging conditions posed by morbid obesity. Despite including only angiograms acquired at maximum tube voltage, and therefore at the performance limits of contemporary fluoroscopic systems, STEP enhanced vessel visibility by expert reader assessment in approximately two-thirds of cases.

These qualitative improvements were corroborated by robust enhancements in objective image quality metrics, including quantitative computations of image contrast, D′, and SNR. Unlike hardware-based solutions, STEP leverages AI and machine learning to postprocess existing images, offering a software-based, retrofit-compatible approach that could be broadly implemented without requiring new capital equipment. By improving vessel visibility, STEP has the potential to increase diagnostic confidence without requiring additional radiation or contrast administration.

The findings of this analysis, which should only be considered hypothesis generating, are limited by unblinded interpretation of images and a lower inter-reader agreement for STEP-processed cineangiograms compared to standard cineangiograms, thereby reflecting inter-reader variability in the assessment of image enhancement across cases. Future prospective, multicenter studies should evaluate STEP’s impact on diagnostic accuracy, procedural outcomes, and radiation exposure, as well as its performance across a broader range of patient body mass indices. In summary, STEP represents a promising AI-developed tool to mitigate imaging challenges in morbidly obese patients.

## CRediT authorship contribution statement

**Samia Mazumder:** Writing – review & editing, Writing – original draft. **Tim Joseph:** Writing – review & editing. **Devraj Sukul:** Writing – review & editing. **Araya Negash:** Writing – review & editing. **Justin Armstrong:** Writing – review & editing. **Lakshmi Rao:** Writing – review & editing. **Najeebullah Bangash:** Writing – review & editing. **Ryan D. Madder:** Writing – review & editing, Writing – original draft, Supervision.

## Declaration of competing interest

Tim Joseph is a consultant for AngioWave Imaging and Boston Scientific and has received speaker honoraria from Abbott Vascular. Devraj Sukul is a consultant for AngioWave Imaging and RapidAI. Araya Negash is a consultant for Infraredx and RapidAI. Ryan D. Madder is an advisory board member for Abbott Vascular, Boston Scientific, Gentuity, Medtronic, and SpectraWAVE; a consultant for Abbott Vascular, Acist, AngioWave Imaging, Boston Scientific, MiRus, Nanoflex Robotics, Orchestra Biomed, Philips, RapidAI, and SpectraWAVE; holds equity interests in AngioWave Imaging and M3D; has received research support from AngioWave Imaging, Microbot Medical, and Nanoflex Robotics; and has received speaker honoraria from Abbott Vascular, Acist, Boston Scientific, and Philips. All other authors reported no financial interests.
